# Ovalbumin Peptide–Selenium Nanoparticles Alleviate Immune Suppression in Cyclophosphamide-Induced Mice: A Combined Transcriptomic and Proteomic Approach to Reveal the Mechanism

**DOI:** 10.3390/foods14132295

**Published:** 2025-06-28

**Authors:** Yingnan Zeng, Qi Yang, Zhiyang Du, Xuanting Liu, Xiaomin Shang, Menglei Xu, Jingbo Liu, Siwen Lyu, Ting Zhang

**Affiliations:** 1 Jilin Provincial Key Laboratory of Nutrition and Functional Food and College of Food Science and Engineering, Jilin University, Changchun 130062, China; 2 Jilin Brewing Technology Innovation Center and College of Food Science and Nutritional Engineering, Jilin Agricultural Science and Technology University, Jilin 132101, China

**Keywords:** ovalbumin peptide, selenium nanoparticles, immunomodulatory activity, transcriptomics, proteomics

## Abstract

Immunocompromise is a growing health concern, and food-derived immunomodulators are expected to serve as a valuable supplement to traditional drug therapies. Ovalbumin peptide (OP) was employed as a stabilizer to prepare OP–selenium nanoparticles (OP-SeNPs), which showed immunomodulatory effects in vitro; however, the effects and underlying mechanisms in vivo were not yet fully understood. This study investigated the immunomodulatory activity of OP-SeNPs in cyclophosphamide (CTX)-induced immunosuppressed mice on immune organs, molecules, and cells, with the underlying mechanism explored by transcriptomic and proteomic studies. The results demonstrated that OP-SeNPs alleviated tissue damage in the spleen and thymus, improved the immunosuppressive state by promoting the secretion of cytokines (IL-1β, IFN-γ, IL-4, and IL-6), immunoglobulins (IgA, IgG, IgM, and sIgA), and promoting the proliferation of splenic lymphocytes. PI3K-Akt, Rap1, p53, PPAR, and Hippo signaling pathways formed an important regulatory network that synergistically influenced immune modulation. OP-SeNPs are potential food-derived immunomodulators, setting the stage for deep exploration of the mechanisms driving their immunomodulatory effects.

## 1. Introduction

Immunocompromise, both in the past and the future, will remain a significant challenge to human health. It is defined as the situation in which the body’s immune system is impaired, leading to reduced immune defense and increased susceptibility to infections and the occurrence of tumors [1]. Various factors can damage the immune system, such as aging, lifestyle factors, stress, and environmental pollution and can lead to immune imbalance [2]. Additionally, diseases and exogenous drugs can contribute to immunosuppression, as seen in patients with primary immunodeficiencies or those undergoing long-term immunosuppressive therapy for cancer treatment [3]. Immunomodulatory drugs such as levamisole hydrochloride (LH) and pidotimod [4] can effectively restore the immune function of immunosuppressed hosts [5]. However, because of their negative impact on the nervous system and gastrointestinal tract, as well as their high cost, these drugs are not ideal for prevention or prolonged use. It is extremely urgent to explore an active compound that is both effective and safe to enhance immunity.

The bioactive peptides derived from food proteins have various functions, such as immunomodulatory, antioxidative, anticancer, and antidiabetic effects [6]. The ovalbumin peptide (OP) derived from egg white has garnered significant interest due to its low toxicity and high activity. Previous studies have demonstrated that OP possessed immunomodulatory effects and was capable of alleviating the bodily damage caused by immunosuppression. Li et al. [7] found that the products obtained by hydrolyzing ovalbumin with alkaline protease and papain could enhance the phagocytic activity in RAW264.7 macrophages (a murine macrophage cell line) and increase the levels of nitric oxide and cytokines, thereby achieving the function of enhancing immunity. Furthermore, a study found that OP reversed the immunosuppressive effects induced by cyclophosphamide (CTX) by enhancing immune organ function and increasing levels of immunoglobulins and cytokines [8]. Therefore, OP is a potential candidate for food-derived immune modulation.

Selenium is an essential trace element that must be obtained from food, playing a key role in human physiological functions, especially in enhancing immune responses and providing antioxidant protection [9]. A new type of selenium nanoparticles (SeNPs) has been developed that not only has a simple preparation method at a low cost, but also possesses higher bioavailability and lower toxicity, which will be a promising candidate for nutritional fortification and therapeutic applications [10]. SeNPs are emerging as a rising star in the field of immunotherapy. Research has demonstrated that SeNPs contribute significantly to activating the body’s innate and adaptive immune responses, exerting immunomodulatory effects to affect the onset of diseases, and serving as an adjunct in chemotherapy [11]. But SeNPs are prone to aggregation and loss of activity because of their high surface free energy [12]. The presence of stabilizers such as peptides can facilitate the self-assembly of SeNPs [13]. Our previous study results indicated that OP, as a stabilizer, formed a composite with SeNPs, referred to as OP-SeNPs, which could promote the production of nitric oxide and enhance phagocytic capacity in RAW264.7 macrophages, demonstrating enhanced immunomodulatory activity [14]. However, the underlying mechanisms of the OP-SeNPs complex in vivo were not yet fully understood.

Although previous studies have preliminarily revealed the biological activities of SeNPs, they are often limited to focusing on single pathways or simple phenotypic observations, which makes it difficult to fully elucidate their mechanisms of action in the complex biological environment. Compared to traditional research methods, transcriptomics and proteomics are critical approaches for analyzing potential mechanisms of action. Some researchers have utilized a zebrafish model, employing transcriptomics and proteomics to investigate the immune regulatory role of SeNPs through their interactions in antioxidant regulation, lipid metabolism, and immune modulation [15]. This study aims to utilize transcriptomics and proteomics to deeply explore the synergistic regulatory signaling pathways induced by OP-SeNPs and thoroughly dissect their regulatory mechanisms at both the transcriptional and translational levels. This approach not only enriches the synergistic regulatory signaling pathways induced by OP-SeNPs but also provides a comprehensive insight into their mechanisms of action.

This study involved the establishment of a CTX-induced immunosuppressed mouse model to investigate whether OP-SeNPs could alleviate immunosuppression in terms of the recovery of immune organs, molecules, and cells, and further analyzed the potential mechanisms of action using transcriptomics and proteomics. This study will lay the groundwork for developing a new food-derived immunomodulatory compound, potentially leading to the enhancement of immune function and overall health.

## 2. Materials and Methods

### 2.1. Materials and Reagents

Ovalbumin, cysteine, CTX, and lipopolysaccharide (LPS) were supplied by Sigma (St. Louis, MO, USA). Papain was obtained from Shanghai Yuanye (Shanghai, China). Penicillin-streptomycin, RPMI-1640 medium, and fetal bovine serum (FBS) were supplied by Gibco (Thermo Scientific, New York, NY, USA). ELISA kits for biomolecule detection were supplied by Shanghai Enzyme-linked Biotechnology (Shanghai, China). LH, concanavalin A (ConA), paraformaldehyde, and MTS cell proliferation kits were supplied by Beijing Solarbio (Beijing, China). Reagents used in the experiment were of analytical grade.

### 2.2. OP-SeNPs Preparation

The preparation method of OP-SeNPs was based on our previous research [14]. Firstly, 5% (*w*/*v*) ovalbumin was incubated at 90 °C for 10 min, hydrolyzed with 3000 U/g papain at pH 6.0, 37 °C for 3 h. The solution was incubated at 90 °C for 10 min, then centrifuged at 9000 r/min for 10 min, and ultrafiltered with a 1 kDa MWCO membrane. The hydrolysates smaller than 1 kDa were freeze-dried to yield OP. Then, 5 mM Na_2_SeO_3_, 2 mM cysteine, and 5 mg/mL OP were combined in a 1:3:1 volume ratio and stirred for 2 h, and ultrafiltered to remove excess Na_2_SeO_3_. The selenium content in OP-SeNPs was determined by ICP-MS (Agilent, Santa Clara, CA, USA) after adding nitric acid to the sample and dissolving it in a microwave sample preparation system (Shanghai Xinyi, Shanghai, China).

### 2.3. Animal Experiment

#### 2.3.1. Animal Treatment

Specific pathogen-free male Balb/c mice (7 weeks) were supplied by Beijing Viton Lihua Laboratory Animal Technology Co. (Beijing, China) and were housed in the Experimental Animal Center of Jilin University’s First Hospital (ethical approval: 20231213-01) at 24 ± 1 °C, following a 12 h light/dark cycle.

Animal experimental design followed the protocol established by Jiang et al. [16]. Following a 7-day adjustment, the mice were divided randomly into 5 groups (*n* = 8): control check (CK); model (M); positive drug (P), LH 40 mg/kg; low dose of OP-SeNPs (OP-SeNPs-L), 200 mg/kg OP + OP-SeNPs (15 μg Se/kg); and high dose of OP-SeNPs (OP-SeNPs-H), 200 mg/kg OP + OP-SeNPs (30 μg Se/kg). An immunosuppressive model was established in mice by intraperitoneal injection of CTX (80 mg/kg) for 3 days [17]. Subsequently, the mice were administered drugs by intragastric gavage for 14 days, while the CK group received an equal volume of water (Figure S1). The mice were euthanized following the approved animal protocols.

#### 2.3.2. Immune Organ Indices

Spleen and thymus were excised, and their weights were measured. Organ index = organ weight (mg)/body weight (g).

#### 2.3.3. Histopathological Analysis

Spleen and thymus were preserved in 4% paraformaldehyde solution for over 24 h, processed into paraffin sections, stained with hematoxylin and eosin (H&E), and then analyzed.

#### 2.3.4. Biochemical Assay

Serum was prepared from the collected blood by centrifuging at 3000 r/min, 4 °C for 15 min. The small intestine tissue was added with PBS, fully homogenized with a homogenizer, and the homogenate was centrifuged at 3000 r/min, 4 °C for 15 min. Serum and the supernatant of homogenized small intestine were separately used for ELISA to determine the biomolecules.

#### 2.3.5. Splenic Lymphocyte Proliferation Assay

The preparation of splenic lymphocytes was carried out under sterile conditions, following the protocol provided by Dai et al. [18]. The spleen was minced until no large pieces of red tissue remained, then filtered through a 70 µm mesh sieve. PBS was added to wash the cells, and the processed sample was centrifuged at 350× *g* for 5 min to remove the supernatant. Red blood cell (RBC) lysis buffer was added and incubated on ice for 5 min. Afterward, the sample was treated with PBS once more and centrifuged at 350× *g* for 5 min. Upon removal of the supernatant, the cells were resuspended in RPMI-1640 medium with 10% FBS.

T lymphocyte and B lymphocyte proliferation assays were separately stimulated with ConA and LPS, respectively [19]. The cells (1.0 × 10^5^ cells/well) were seeded in 96-well plates and incubated with 100 μL ConA (5 μg/mL) or 100 μL LPS (10 μg/mL) for 72 h. Following the incubation, the cells were treated with MTS (20 μL) and incubated at 37 °C for 2 h [20]. A microplate reader (Biotek, Winooski, VT, USA) was applied to measure absorbance at 490 nm:

$$\mathrm{Cell}\;\mathrm{proliferation}\;\mathrm{rate}\;(\%)=\frac{{\mathrm{OD}}_{\mathrm{sample}}-{\;\mathrm{OD}}_{\mathrm{blank}}}{{\mathrm{OD}}_{\mathrm{negative}}-{\mathrm{OD}}_{\mathrm{blank}}}\;\times \;100$$

#### 2.3.6. Hematological Analysis

Whole blood was collected from the mice into anticoagulation tubes, and the numbers of peripheral white blood cells (WBC), RBC, as well as the hemoglobin (HGB) concentration, were measured using a fully automated hematology analyzer (Genrui, Shenzhen, Guangdong, China).

### 2.4. Transcriptomic Studies

Transcriptomic studies were performed following the method described in the literature [21]. Total RNA was isolated from the spleen, and mRNA was selectively enriched using Oligo (dT) beads. The cDNA synthesis was performed with the SuperScript kit and random hexamer primers. The cDNA was then end-repaired, phosphorylated, and an ‘A’ base was incorporated following Illumina’s library preparation guidelines. Libraries were size selected for 300 bp fragments via gel electrophoresis and amplified by PCR. After quantification with the Qubit 4.0 system, the paired-end RNA-seq library was sequenced on a NovaSeq X Plus. Then raw reads were processed using fastp, and cleaned reads were aligned to the reference genome with HISAT2 and assembled with StringTie.

Different expression analysis was carried out with DESeq2 (version 1.26.0) software at Majorbio Cloud Platform, and the screening criteria for significant expression differences were fold change (FC) ≥ 1.5 and *p* adjust < 0.05. Finally, Kyoto Encyclopedia of Genes and Genomes (KEGG) pathways (http://www.genome.jp/kegg/, 6 September 2023) enrichment analysis was carried out by KOBAS.

### 2.5. Proteomic Studies

Proteomic studies were based on the protocol outlined by Lou and Shui [22]. The spleen was suspended in protein lysis buffer and lysed by a high-flux tissue grinding instrument. The sample was centrifuged at 12,000× *g* for 30 min, after which the supernatant was harvested. Next, 100 μg protein was resuspended in triethylammonium bicarbonate buffer (TEAB), and tris (2-carboxyethyl) phosphine was added and kept for 1 h at 37 °C. Iodoacetamide (IAM) was subsequently added to the sample, followed by a 40 min incubation away from light. After centrifugation, the pellet was harvested and re-dissolved in TEAB. Trypsin was added to the sample, followed by overnight incubation at 37 °C. The peptides were then desalted with HLB, and peptide quantification was carried out using NanoDrop ONE (Thermo Fisher Scientific, New York, NY, USA). Finally, the peptides were analyzed using data-independent acquisition (DIA) relative quantification assay, with a Vanquish Neo (Thermo, New York, NY, USA) paired with an Orbitrap Astral mass spectrometer (Thermo, New York, NY, USA). Thermo Xcalibur 4.7 (Thermo, New York, NY, USA) was used as the data acquisition.

Differentially expressed proteins (DEPs) were identified at Majorbio Cloud Platform (https://cloud.majorbio.com), using FC ≥ 1.5 and *p* value < 0.05 as thresholds. Functional annotation of the identified proteins was conducted via KEGG pathway analysis.

### 2.6. Statistical Analysis

The normality of the data was tested by the Shapiro–Wilk test, and the variances were tested by the Brown–Forsythe test. ANOVA analysis with Tukey’s test was applied using SPSS 21.0 software, with all experiments performed at least in triplicate. Differences with *p* < 0.05, *p* < 0.01, and *p* < 0.001 were considered statistically significant.

## 3. Results and Discussion

### 3.1. OP-SeNPs Repaired Damage to the Immune Organs

In this study, the Se content of OP-SeNPs was measured by ICP-MS, reaching 8.94%. Previous studies have shown that SeNPs at 20–60 μg Se/kg were shown to protect mice against DSS-induced ulcerative colitis [23], while tuna polypeptide-SeNPs at 18.23–72.92 μg Se/kg enhanced the phagocytic activity of the mononuclear phagocytic system and increased the levels of immunological molecules in mice [16]. Based on these measurements and prior findings, two experimental dosages were selected after converting for body surface area [24]: OP-SeNPs-L at 15 μg Se/kg, which is comparable to the recommended daily selenium intake for adults [25], and OP-SeNPs-L at 30 μg Se/kg, a dose that is well below the tolerable upper intake level of 400 μg for adults [26].

**Figure 1:**
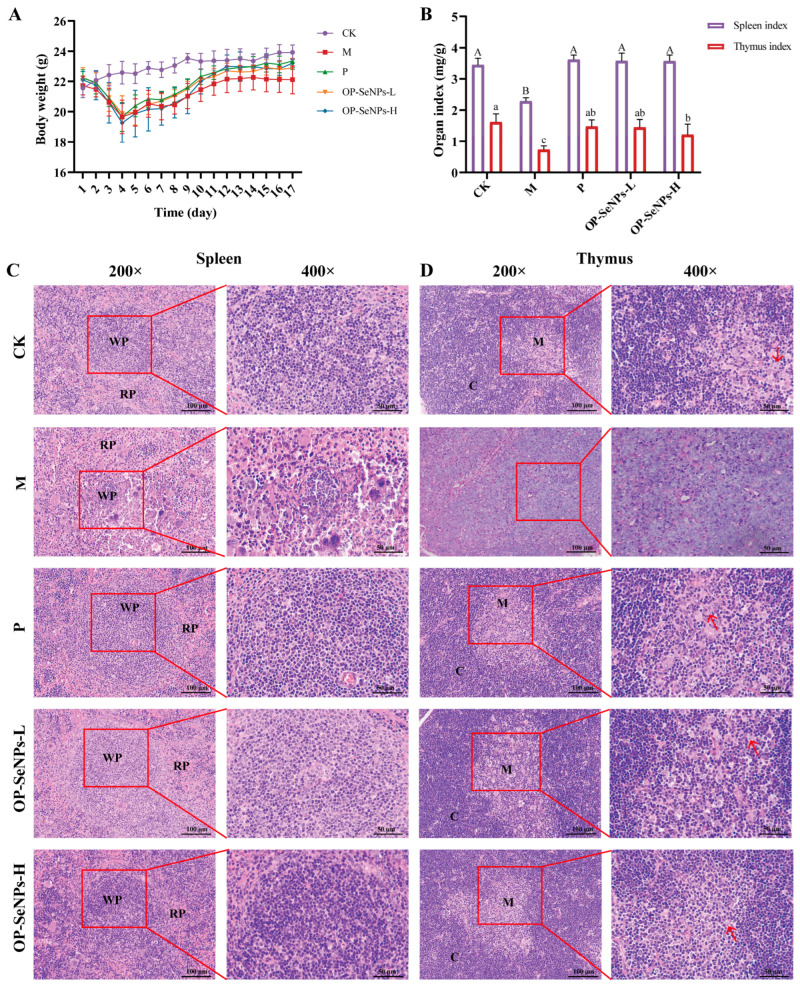
(**A**) Body weight, (**B**) immune organ indices, (**C**) histopathological analysis of spleen (200× and 400×), and (**D**) thymus (200 × and 400 ×) in mice. Column data marked without the same superscripts (a–c, A,B) differed significantly (*p* < 0.05). Red pulp (RP), white pulp (WP), cortex (C), medulla (M), and thymic epithelial cells (arrow).

To investigate the immune-modulating effects of OP-SeNPs, an immunosuppression model was established using mice treated with CTX. As shown in Figure 1A, after three consecutive days of intraperitoneal CTX injection, the body weight in the drug groups decreased rapidly compared with the CK group on the third and fourth day. CTX, a commonly used chemotherapy drug in clinical practice, is metabolized to generate phosphoramide mustard, which exhibits relatively strong immunosuppressive effects [16]. Consequently, the reduction in body weight during this period was primarily due to the toxic side effects of CTX. As the experiment progressed, after the intragastric gavage of OP-SeNPs, the OP-SeNPs group showed an upward trend over the M group. At the beginning of the seventh day, the OP-SeNP-L group showed an obvious trend of rapid rise in body weight, and the rise was higher than that of the M group. This suggested that OP-SeNPs alleviated the weight loss of mice, implying a reversal of the immune suppression effects caused by CTX.

The spleen and thymus, as central immune organs [27], are the sites of immune cell production, proliferation, differentiation and maturation, and they are essential for maintaining the immune defense system [28]. The spleen and thymus indices act as indicators of peripheral immune organs. Figure 1B presents a considerable decrease in the indices of the spleen and thymus that was observed in the M group as opposed to the CK group (*p* < 0.05). CTX damaged the immune system, causing the spleen and thymus to undergo atrophy, and a reduction in the indices of the immune organs [29]. Furthermore, compared with the M group, the indices of the spleen and thymus increased following administration of LH and OP-SeNPs (*p* < 0.05). And no notable difference was observed in the immune organ indices in the OP-SeNPs-L and OP-SeNPs-H groups. The results suggested OP-SeNPs helped repair immune organ damage in CTX-induced immunosuppressed mice.

To evaluate the effects of OP-SeNPs on tissue repair in the spleen and thymus, histopathological examination of both organs was examined using H&E staining. As shown in Figure 1C (200× and 400×), the spleen in the CK group exhibited normal histological morphology, with the parenchyma showing distinct red pulp and white pulp with clear boundaries between them. In contrast, the atrophy of the white pulp and the blurred boundaries between the white and red pulp in the M group were observed. The histological features were characteristic of the immunosuppression model, suggesting potential impairment of the spleen’s immune system. Compared with the M group, after LH and OP-SeNPs treatment, the damage was effectively alleviated; the boundary between the red and white pulp was clear, and the lymphocyte density was restored. Since the red and white pulp in the spleen is rich in T and B lymphocytes, which perform distinct functions and cooperate with each other during immune response, the spleen is vital to adaptive immunity [30]. A paper reported similar results, demonstrating that whey protein isolate-galacto-oligosaccharide conjugates restored spleen function in immunosuppressed mice [17].

The H&E results of the thymus were shown in Figure 1D (200× and 400×). Compared with the CK group, the thymus in the M group showed pathological changes, with the light staining of pathological slides likely due to the low density of lymphocytes. There was no obvious boundary between the medulla and cortex, and the central cortex in the M group was nearly absent. These results indicated that CTX induced pathological damage to the thymus tissue [31]. Compared with the M group, administration of OP-SeNPs resulted in a clearer boundary between the cortex and medulla. The cortical cells were closely connected again, and adjacent thymic epithelial cells formed a network. These findings suggested that OP-SeNPs alleviated the damage caused by CTX to the spleen and thymus in immunosuppressed mice. And similar findings were reported that monkfish roe peptides attenuated thymus injury in immunosuppressed mice [32].

### 3.2. OP-SeNPs Regulated the Cytokine and Immunoglobulin Secretion

**Figure 2:**
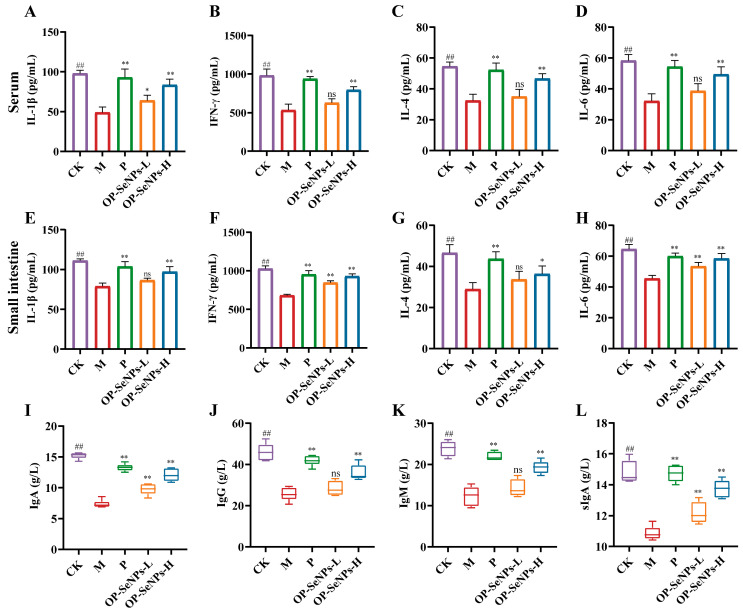
(**A**) IL-1β, (**B**) IFN-γ, (**C**) IL-4, and (**D**) IL-6 levels in the serum; (**E**) IL-1β, (**F**) IFN-γ, (**G**) IL-4, and (**H**) IL-6 levels in the small intestine; (**I**) IgA, (**J**) IgG, and (**K**) IgM levels in the serum; (**L**) secretory IgA (sIgA) levels in the small intestines of mice. CK vs. M group: ## *p* < 0.01; P, OP-SeNPs-L, and OP-SeNPs-H vs. M group: ** *p* < 0.01, * *p* < 0.05, and ns means no significant difference.

Cytokines are small molecules secreted by immune cells in response to stimulation, which bind to specific receptors to exert immune regulatory effects [33]. Figure 2 presents the secretion levels of cytokines and immunoglobulins in the serum and small intestine. All levels of cytokines and immunoglobulins significantly declined in the M group compared to the CK group (*p* < 0.01), exhibiting that CTX impaired the immune system [34]. Under treatment with OP-SeNPs-L, the levels of IL-1β in the serum were considerably increased by 30.60%, and the levels of IFN-γ and IL-6 in the small intestines were notably increased by 24.17% and 17.58% compared with the M group (Figure 2A–H). Meanwhile, cytokine levels in the OP-SeNPs-H group were higher than the OP-SeNPs-L group, approaching those of the CK group. Cellular immunity is enhanced via helper T1 (Th1) cells producing IL-1β and IFN-γ, while humoral immunity is enhanced via Th2 cells producing IL-4 and IL-6 [35]. OP may exert its immunomodulatory effects by activating Th cells, which regulate the secretion of cytokines to enhance cellular and humoral immunity.

Immunoglobulins are produced by activated B cells and serve as important effector molecules in mediating humoral immunity [19]. Figure 2I–K present, in contrast to the CK group, the M group exhibiting a considerable decline in the levels of IgA, IgG, and IgM (*p* < 0.01). Compared to the M group, OP-SeNPs-L markedly increased the secretion level of IgA (*p* < 0.01), while OP-SeNPs-H promoted a stronger increase in immunoglobulin levels, with the contents of IgA, IgG, and IgM being 12.05 g/L, 35.92 g/L, and 19.33 g/L, respectively. The secretion of immunoglobulins was consistent with the cytokine results, both indicating that OP-SeNPs enhanced humoral immunity. Secretory IgA (sIgA) is produced in the intestine, which is the largest immune organ of the human body, where it functions as the initial barrier against microbial invasion. The small intestinal sIgA levels in the OP-SeNPs-H group increased by 26.48% against the M group (Figure 2L). Accordingly, it can be speculated that OP-SeNPs-H promoted sIgA secretion by plasma cells in the intestinal lamina propria, thereby enhancing the front-line defense at the mucosal surface and protecting both intestinal and systemic immunity [36]. Therefore, the findings demonstrated that OP-SeNPs-H notably enhanced the production of cytokines and immunoglobulins, strengthening immune responses and effectively reversing the immunosuppression induced by CTX.

The potential immunomodulatory properties of OP were explored. As shown in Figure S2A, OP can alleviate the damage induced by CTX. Compared to the OP group, a significant increase in the levels of IFN-γ, IL-4, IgA, IgG, and IgM in the serum of the OP-SeNPs-H group was observed (Figure S2B–F). The incorporation of SeNPs increased the cytokine levels in the mice, leading to a more significant reversal of immune suppression and thereby improving the efficacy of OP-SeNPs-H in immune regulation. The observed effect was suggested to be associated with the ability of SeNPs to activate immune responses, attributed to their biological activity and nanostructural characteristics [11], and SeNPs were also considered potential immune modulators.

### 3.3. OP-SeNPs Modulated Immune Cell Function

**Figure 3:**
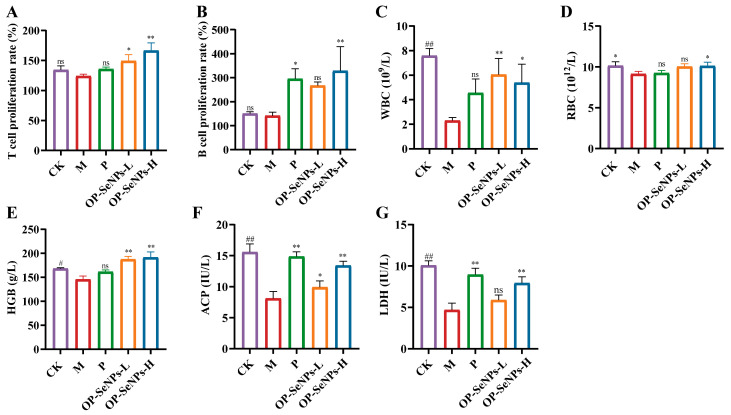
(**A**) Splenic T cell proliferation and (**B**) B cell proliferation; (**C**) the count of white blood cells (WBC), (**D**) red blood cells (RBC), and (**E**) hemoglobin (HGB) concentration of mice; (**F**) the activities of acid phosphatase (ACP) and (**G**) lactate dehydrogenase (LDH) in the serum of mice. CK vs. M group: ## *p* < 0.01, # *p* < 0.05; *p*, OP-SeNPs-L, and OP-SeNPs-H vs. M group: ** *p* < 0.01, * *p* < 0.05, and ns means no significant difference.

The activation of cellular and humoral immune responses heavily relies on lymphocyte proliferation [37]. To assess the proliferative ability of splenic lymphocytes (including T and B cells), isolated splenocytes were stimulated with ConA/LPS, and the T and B cell proliferation rate was measured using MTS assay (Figure 3A,B). OP-SeNPs-H substantially promoted T and B cell proliferation compared with the M group (*p* < 0.01), and the increased number of lymphocytes enabled better antigen recognition, thus activating the adaptive immune system. This result was consistent with the mediation of immunity as indicated by the results of cytokines and immunoglobulin assays mentioned above.

White blood cells (WBC) are key indicators in defending and protecting the body. Changes in WBC levels are important indicators for diagnosing diseases associated with weakened immune function [38,39]. Figure 3C–E present significantly lower levels (*p* < 0.05) of peripheral WBC, RBC, and HGB observed in the M group compared to the CK group, which demonstrated the mice were in an immunosuppressive state. In comparison with the M group, the OP-SeNPs-H group showed a remarkable increase in the levels of WBC, RBC, and HGB (*p* < 0.05), suggesting that OP-SeNPs alleviated the immunosuppression. Similar findings have been found that three types of polysaccharides (from Dendrobium officinale, Aloe, and Konjac) recovered immune suppression by increasing the levels of WBC, RBC, and HGB [40].

The activation ability of macrophages is, to some extent, reflected by the activities of acid phosphatase (ACP) and lactate dehydrogenase (LDH) [41]. Therefore, we measured the activities of these two enzymes in the serum using ELISA kits to evaluate the functional state of macrophages. A notable reduction (*p* < 0.01) in the levels of ACP and LDH was observed in the M group treated with CTX (Figure 3F,G). Compared to the M group, the activities of ACP and LDH in OP-SeNPs-H increased by 65.08% and 68.98%, respectively. OP-SeNPs-H enhanced macrophage activation, which was related to the activation of innate immunity. To summarize, OP-SeNPs promoted immune cell proliferation, thereby exerting immunomodulatory effects.

### 3.4. OP-SeNPs Enhanced Antioxidant Activity

**Figure 4:**
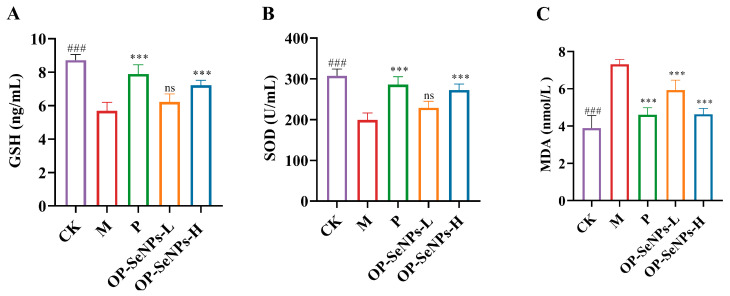
(**A**) GSH, (**B**) SOD, and (**C**) MDA levels in the serum of mice. CK vs. M group: ### *p* < 0.001; P, OP-SeNPs-L, and OP-SeNPs-H vs. M group: *** *p* < 0.001, and ns means no significant difference.

CTX induces oxidative stress, leading to oxidative damage to cells and organisms. To evaluate the antioxidative properties of OP-SeNPs, the determination was carried out on antioxidant molecules such as glutathione (GSH) and superoxide dismutase (SOD), as well as the lipid peroxidation product malondialdehyde (MDA) [42]. Compared to the CK group (Figure 4A–C), the serum levels of GSH and SOD in the M group exhibited a significant reduction, and the MDA levels were significantly elevated (*p* < 0.001). In contrast to OP-SeNPs-L, OP-SeNPs-H demonstrated a superior ability to increase the levels of GSH and SOD and reduce the MDA levels. It proved that OP-SeNPs can protect against oxidative damage in a dose-dependent manner, likely due to the strong antioxidant properties of selenium, which may activate the antioxidant defense system and help alleviate CTX-induced damage. This finding was consistent with the results, where Se-enriched *G. frondosa* polysaccharide substantially raised the production of GSH-Px, SOD, and CAT, thereby protecting tissues from oxidative stress-induced damage [19].

### 3.5. Transcriptomic Analysis of the Spleen

#### 3.5.1. Transcriptomic Principal Component Analysis

**Figure 5:**
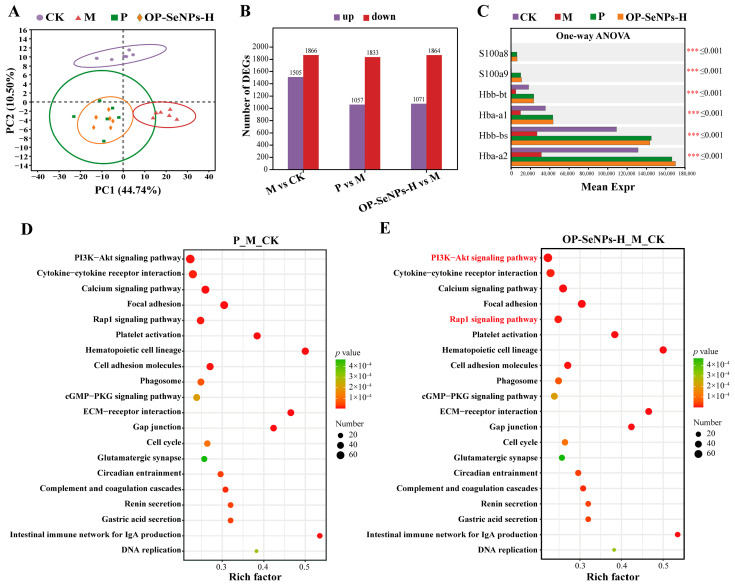
(**A**) Spleen transcriptomic analysis: principal component analysis (PCA) of genes, (**B**) the number of differentially expressed genes (DEGs), (**C**) multiple comparison test of genes, (**D**) KEGG enrichment analysis of P_M_CK gene set (top 20), and (**E**) KEGG enrichment analysis of OP-SeNPs-H_M_CK gene set (top 20). *** *p* < 0.001. P_M_CK gene set: M_CK_up vs. P_M down and M_CK_down vs. P_M up; OP-SeNPs-H_M_CK gene set: M_CK_up vs. OP-SeNPs-H_M down and M_CK_down vs. OP-SeNPs-H_M up.

To clarify the mechanism of the immunomodulatory effects of OP-SeNPs, the total RNA of the spleen was sequenced using transcriptomic methods. The principal component analysis (PCA) of the gene analysis showed good repeatability of the samples within each group (Figure 5A). CTX-induced immunosuppression caused significant gene expression changes between the CK and M groups. The OP-SeNPs-H group, similar to the P group, effectively improved these gene expression alterations. It may play a positive role in alleviating gene abnormalities associated with immune suppression.

#### 3.5.2. Analysis of Differentially Expressed Genes

Compared with the CK group (Figure 5B), a total of 3371 genes were differentially expressed in the M group, including 1505 up-regulated and 1866 down-regulated. And compared with the M group, differentially expressed genes (DEGs) in the P group were a total of 2890, including 1057 up-regulated and 1833 down-regulated. OP-SeNPs-H DEGs were a total of 2935, including 1071 up-regulated and 1864 down-regulated. The multiple comparison test was shown in Figure 5C. The HGB genes, including Hbb-bt, Hba-a1, Hbb-bs, and Hba-a2 were up-regulated by OP-SeNPs-H and LH treatment. It was consistent with the result of the increase in HGB concentration from the hematological analysis in the OP-SeNPs-H group, suggesting that HGB played an important role in cell signaling and immunomodulation.

#### 3.5.3. KEGG Enrichment Analysis of Differentially Expressed Genes

As shown in Figure 5D,E, the top 20 KEGG enrichment was performed by analyzing gene differences to deeply investigate the enrichment of relevant biological pathways, thereby revealing the molecular mechanism of immunomodulation by OP-SeNPs-H in immunosuppressed mice. The filtering criterion for DEGs was FC ≥ 1.5 and *p* adjust < 0.05, with regulation (up/down) used to obtain the gene set, as such the P_M_CK set was composed of M_CK_up vs. P_M down and M_CK_down vs. P_M up, and the OP-SeNPs-H_M_CK set was composed of M_CK_up vs. OP-SeNPs-H_M down and M_CK_down vs. OP-SeNPs-H_M up. The results exhibited signaling pathways such as Phosphoinositide 3-kinase (PI3K)-Akt, Calcium, Ras-associated protein-1 (Rap1), and cGMP-PKG signaling pathways in the P_M_CK and OP-SeNPs-H_M_CK set. Interestingly, as shown in Figure 5E, the KEGG analysis in the OP-SeNPs-H_M_CK set revealed enrichment in several pathways, including cytokine–cytokine receptor interaction, ECM-receptor interaction, focal adhesion, and cell adhesion molecules. Additionally, platelet activation, Hematopoietic cell lineage, and Complement and coagulation cascades were also found to be enriched. The involvement of Complement and coagulation cascades implied that the immunomodulatory role played by OP-SeNPs-H might be related to the innate immune system [43]. Therefore, it was speculated that OP-SeNPs-H could not only stimulate hematopoietic stem cells, prompting them to stimulate differentiation into various types of WBC, but also enhance extramedullary hematopoiesis, promote the growth and maturation of blood cells, and thereby achieve immunomodulatory effects [40].

### 3.6. Proteomic Analysis of the Spleen

#### 3.6.1. Proteomic Principal Component Analysis

**Figure 6:**
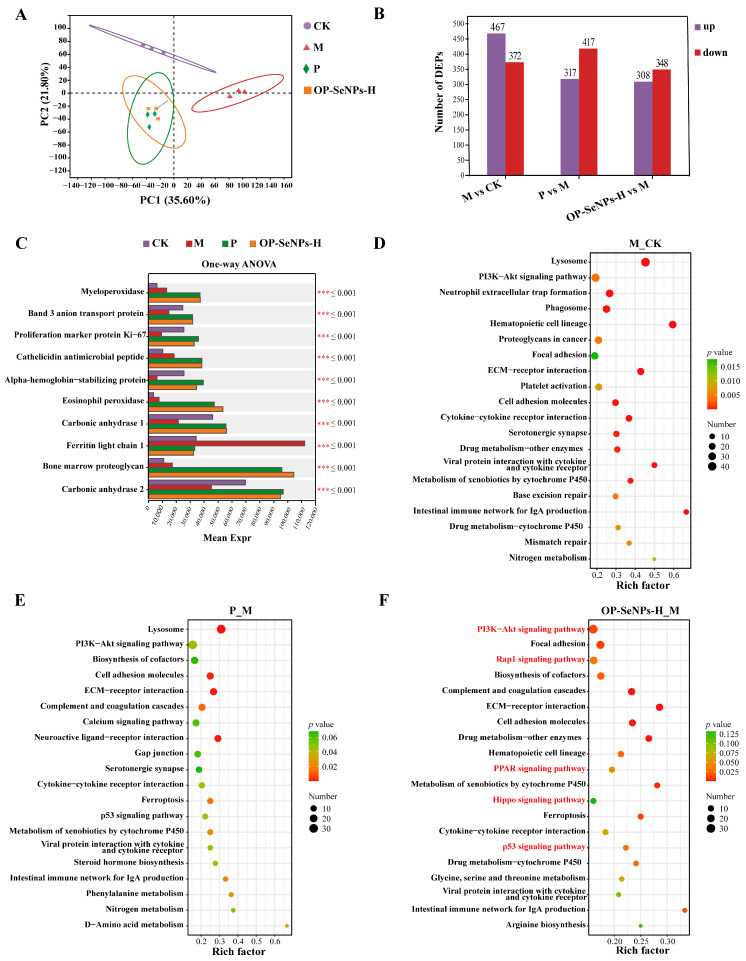
(**A**) Spleen proteomic analysis: principal component analysis (PCA) of proteins, (**B**) the number of differentially expressed proteins (DEPs), (**C**) multiple comparison test of proteins, (**D**) KEGG enrichment analysis of M_CK protein set (top 20), (**E**) KEGG enrichment analysis of P_M protein set (top 20), and (**F**) KEGG enrichment analysis of OP-SeNPs-H_M protein set (top 20). *** *p* < 0.001. M_CK protein set: M vs. CK; P_M protein set: P vs. M; OP-SeNPs-H_M protein set: OP-SeNPs-H vs. M.

To elucidate the impact of OP-SeNPs on protein expression in immunosuppressed mice, the proteome of the spleen was comprehensively resolved using DIA-relative quantification. PCA was used to clarify the difference in distribution between samples (Figure 6A). The PC1 and PC2 dimensions clearly distinguished the CK and M groups, with CTX intervention causing significant alterations in protein expression. Both the P and OP-SeNPs-H groups showed trends toward the CK group, indicating improvement in protein expression. OP-SeNPs-H specifically drove differential protein expression. This trend aligned with the transcriptomic PCA results.

#### 3.6.2. Analysis of Differentially Expressed Proteins

Compared with the CK group (Figure 6B), a total of 839 proteins were differentially expressed in the M group, including 467 up-regulated and 372 down-regulated. Compared with group M, DEPs in the P group were a total of 734, including 317 up-regulated and 417 down-regulated. DEPs in the OP-SeNPs-H group were a total of 656, including 308 up-regulated and 348 down-regulated. In the comparative analysis of DEPs in multiple groups (Figure 6C), compared with the M group, the proteins showed a considerable rise (*p* < 0.001) in the OP-SeNPs-H group associated with immunization, including myeloperoxidase, cathelicidin antimicrobial peptide, and eosinophil peroxidase. Myeloperoxidase was a marker of activated neutrophils, and its level and activity reflected the function and state of neutrophils [44]. It catalyzed the production of hypochlorous acid to defend against the attack of pathogens, thus contributing to innate immunity [45]. Cathelicidin antimicrobial peptide was primarily produced by immune cells, which, upon processing, generated the mature LL-37 peptide that possessed antimicrobial and immune function, serving as a key component of innate immune defense [46]. Eosinophil peroxidase is a granule protein uniquely expressed in eosinophils, which can exert both anti-inflammatory and pro-inflammatory effects, and can also act as a cationic toxin to kill parasites [47].

#### 3.6.3. KEGG Enrichment Analysis of Differentially Expressed Proteins

The top 20 KEGG enrichment was applied to evaluate the enrichment of DEPs in biological pathways, aiming to uncover the molecular mechanism of OP-SeNPs-H in alleviating immunosuppression. As shown in Figure 6D–F, DEPs were mainly enriched in the immune system, signal transduction, signaling molecules and interactions, cellular processes, and amino acid metabolism. Some immune system-related pathways were enriched in the OP-SeNPs-H_M protein set (Figure 6F), including Complement and coagulation cascades, Hematopoietic cell lineage, and Intestinal immune network for IgA production, which was consistent with the increase in sIgA secretion levels detected in the small intestine. Concurrently, based on the analysis of KEGG enrichment in transcriptomics, it suggested that OP-SeNPs-H was associated with the Intestinal immune network for IgA production and effectively enhanced the intestinal mucosal barrier function.

In the KEGG enrichment analysis, the key genes driving the PI3K-Akt pathway primarily included growth factor (GF), receptor tyrosine kinase (RTK), Ras, cytokine, cytokine receptor, extracellular matrix (ECM), G-protein-coupled receptors (GPCRs), pH domain leucine rich repeat protein phosphatase (PHLPP), and forkhead box O (FOXO), with genes being either up-regulated or down-regulated (Figure S3).

#### 3.6.4. Analysis of Key Signaling Pathways

The DEPs in the OP-SeNPs-H_M protein set were predominantly associated with several important signaling pathways (Figure 6F). The PI3K-Akt signaling pathway showed the highest number of enriched proteins, which was also consistent with the transcriptomic data. As a member of the lipid kinase family, PI3K regulates a broad spectrum of intracellular processes, with its activation of the PI3K-Akt pathway being pivotal in regulating cellular functions like proliferation, growth, and survival [48]. And the PI3K-Akt pathway could activate RAW264.7 macrophages, exhibiting immune regulatory activity [49], and also activate T lymphocytes, promoting the differentiation of Th cells, including both Th1 and Th2 subsets [50]. The similar results that the protein isolated from *Pleurotus eryngii* (PEP 1b) exerted immunomodulatory effects in macrophages through the Rap1 and PI3K-Akt signaling pathways using the iTRAQ approach [51]. The key proteins involved in driving the PI3K-Akt pathway primarily included GF, RTK, cytokine receptors, ECM, GPCRs, PI3K, and endothelial nitric oxide synthase (eNOS) (Figure S4).

The PI3K-Akt, Rap1, p53, Hippo, and PPAR signaling pathways in the OP-SeNPs-H_M protein set were enriched in KEGG. These signaling pathways significantly contributed to the regulation of immune activation, such as T cell differentiation, migration, and homing, and were closely interconnected with the PI3K-Akt signaling pathway. In the Rap1 signaling pathway, guanine nucleotide exchange factors (GEFs) regulate the exchange of GDP for GTP in the Rap1-GDP complex, which leads to the recruitment of effector molecules to the vicinity of the cell membrane and the activation of the PI3K-Akt signaling pathway [52]. The crosstalk between the P13K-Akt and Hippo signaling pathways exerts a combined effect on cell proliferation and apoptosis, and is of great significance for maintaining cellular homeostasis, normal development of the organism, and disease prevention and control [53]. The PI3K-Akt and p53 signaling pathways interact in a dependent manner, influencing upstream and downstream effector molecules to control cell proliferation and apoptosis. For example, the p53-dependent mechanism induced nuclear Akt activation, which had an impact on cancer [54]. Furthermore, peroxisome proliferator-activated receptors (PPAR) are crucial in T cell migration and homing, with the PPARγ subtype promoting the process of monocyte differentiation into macrophages [55].

The PI3K-Akt, Rap1, p53, Hippo, and PPAR signaling pathways formed a complex regulatory network in various cellular physiological processes. They were interconnected and regulated through multiple molecular mechanisms, synergistically acting on the proliferation and differentiation of immune cells, and contributing significantly to the process of immune regulation.

## 4. Conclusions

In conclusion, OP-SeNPs exhibited effective immunomodulatory effects in reversing CTX-induced immunosuppression in mice. OP-SeNPs alleviated the tissue damage of the spleen and thymus, increased the secretion levels of cytokines and immunoglobulins, exhibited antioxidant activity, and promoted splenic lymphocyte proliferation. The KEGG enrichment analysis based on transcriptomics and proteomics revealed that OP-SeNPs could trigger multiple signaling pathways, including the PI3K-Akt, Rap1, p53, PPAR, and Hippo signaling pathway, to improve immune function. These pathways formed an interconnected regulatory network that regulated immune cell proliferation, differentiation, and overall immune regulation.

SeNPs at low doses under supranutritional levels exhibited either non-toxicity or low toxicity, which was even lower than that of organic Se-methylselenocysteine [56]. Other research has shown that, when administered continuously for 14 days in rats, 0.2 mg Se/kg had positive health effects, while a higher dose of 8 mg Se/kg increased alkaline phosphatase and alanine aminotransferase levels, causing damage to the liver, lung, and thymus, and potentially leading to chronic toxicity [57]. However, in long-term safety studies, it was found that 24 weeks of SeNPs (50 μg Se/kg) exacerbated atherosclerotic lesions in an apolipoprotein E-deficient mouse model [58]. In addition, the Se level from OP-SeNPs-L is close to the recommended daily intake, while the Se level from OP-SeNPs-H, although below the tolerable upper intake level, still requires further investigation into the potential risks of long-term consumption in animal experiments. Based on the results from the mouse model, this research provides preliminary support for the potential development of bioactive peptide-based products in supporting immune health and preventing immunocompromised conditions.

## Data Availability

The original contributions presented in the study are included in the article and Supplementary Materials; further inquiries can be directed to the corresponding authors.

## Supplementary Materials

The following supporting information can be downloaded at: https://www.mdpi.com/article/10.3390/foods14132295/s1. Figure S1: Schematic of the animal experimental protocol and drug administration. Figure S2: (A) Histopathological analysis of spleen (200× and 400×) in mice; (B) IFN-γ, (C) IL-4, (D) IgA, (E) IgG, and (F) IgM in the serum. Figure S3: Key genes in the PI3K-Akt signaling pathway. Figure S4: Key proteins in the PI3K-Akt signaling pathway.
