# Supplementary material for: Ovalbumin Peptide–Selenium Nanoparticles Alleviate Immune Suppression in Cyclophosphamide-Induced Mice: A Combined Transcriptomic and Proteomic Approach to Reveal the Mechanism
Source: Foods. 2025 Jun 28;14(13):2295. doi: 10.3390/foods14132295 (PMC12248530; doi:10.3390/foods14132295)
Supplement: Supplementary file 1 [file foods-14-02295-s001.zip › foods-3662526-supplementary.pdf]

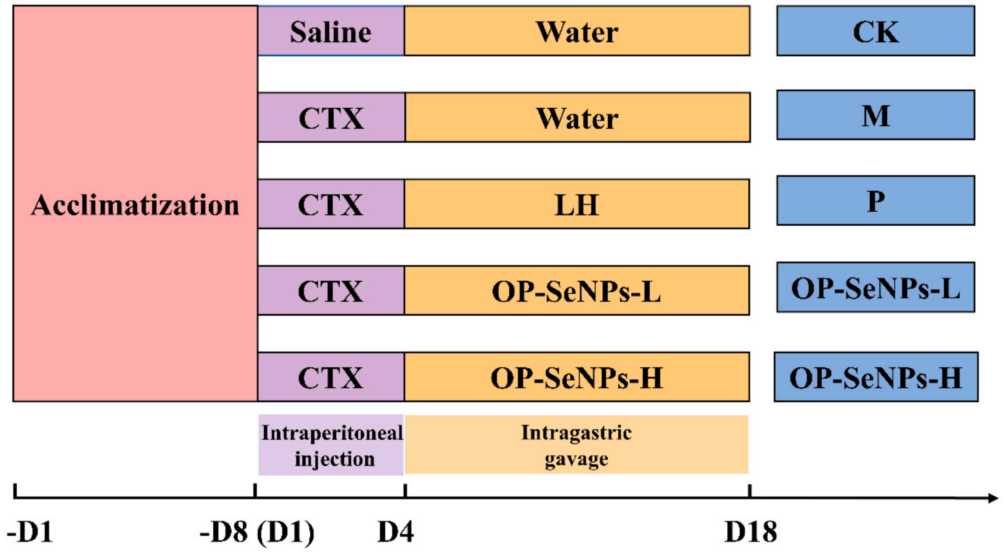

Figure S1. Schematic of the animal experimental protocol and drug administration. Cyclophosphamide (CTX), levamisole hydrochloride (LH).

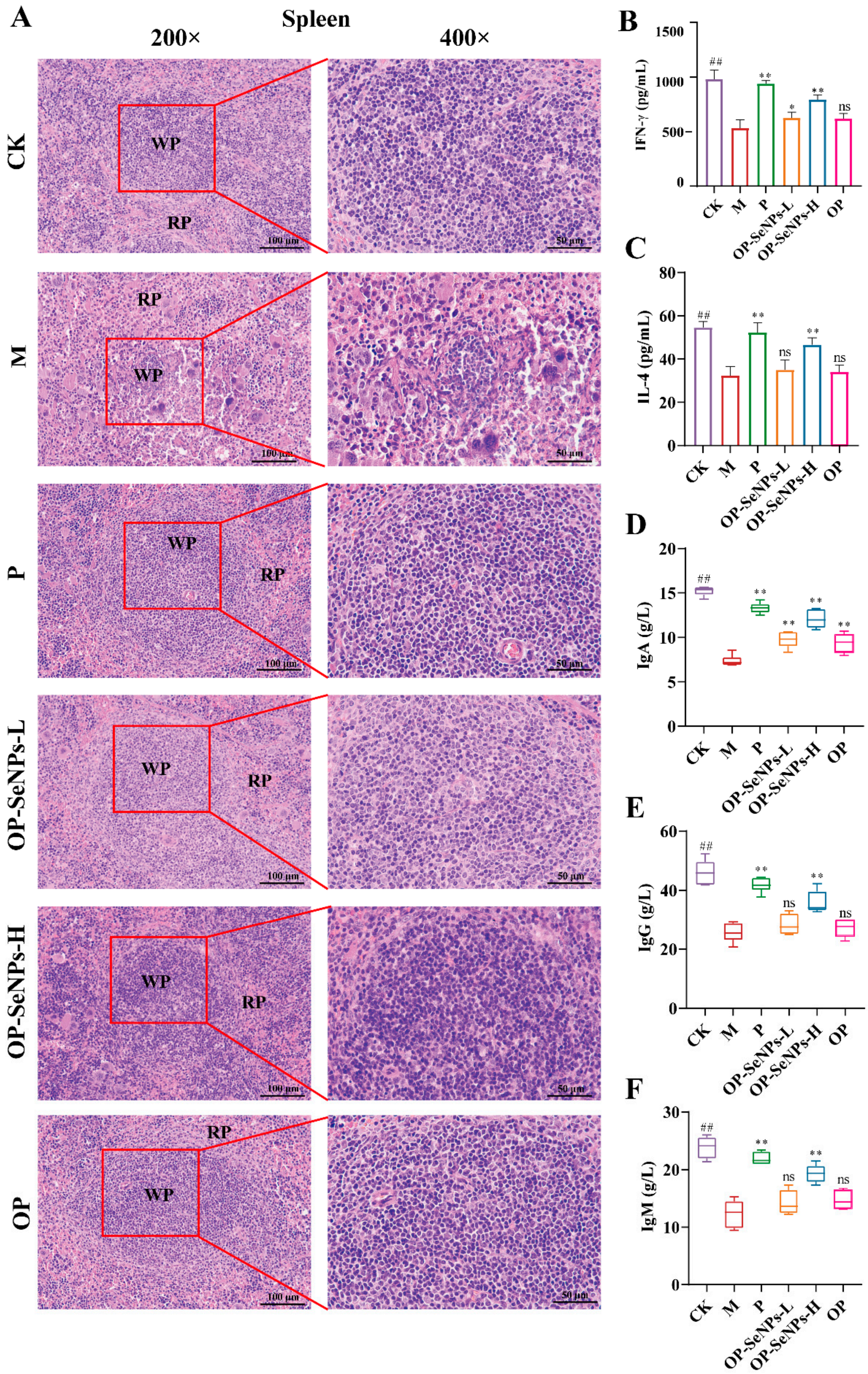

Figure S2. (A) Histopathological analysis of spleen (200 × and 400 ×) in mice; (B) IFN-γ, (C) IL-4, (D) IgA, (E) IgG, and (F) IgM in the serum. Ovalbumin peptide (OP):

200 mg/kg OP. Red pulp (RP), white pulp (WP). CK vs M group: ## $p < 0.01$; P, OP-SeNPs-L, OP-SeNPs-H, and OP vs M group: ** $p < 0.01$, * $p < 0.05$, and ns means no significant difference.

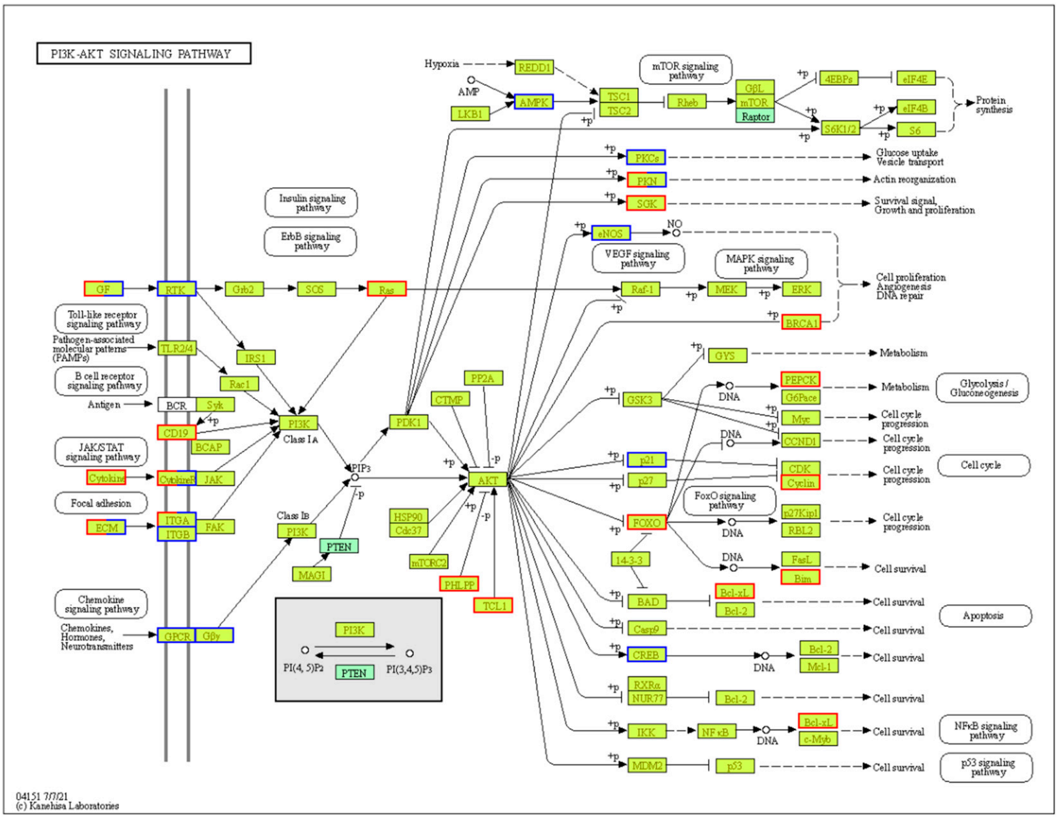

Figure S3. Key genes in the PI3K-Akt signaling pathway. Red (Up-regulated genes), blue (Down-regulated genes), and red + blue (Up + down-regulated genes).

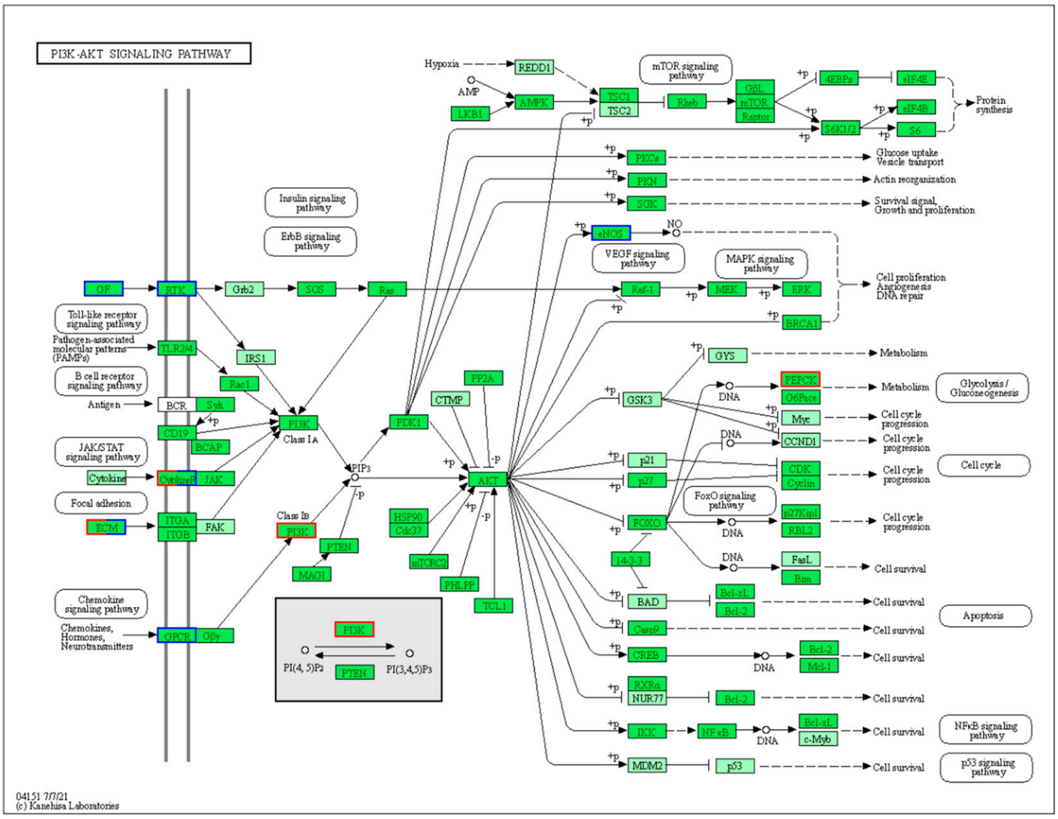

Figure S4. Key proteins in the PI3K-Akt signaling pathway. Red (Up-regulated proteins), blue (Down-regulated proteins) and red + blue (Up + down-regulated proteins).
